# Evaluation of Geophagy Clay Capacity in Adsorbing Cd^2+^ and Pb^2+^ for Water Treatment in Southeast Nigeria

**DOI:** 10.1155/2020/4421117

**Published:** 2020-09-12

**Authors:** T. M. Osobamiro, E. T. Ademuyiwa, O. M. Ajibade, A. S. Hashimi

**Affiliations:** Department of Chemical Sciences, Olabisi Onabanjo University, P. M. B. 2002, Ago-Iwoye, Nigeria

## Abstract

Geophagy clay has been used in tropical regions as gastrointestinal protector for adsorbing toxins in human body, but it was rarely used in adsorbing heavy metals contaminants in water. This study determines elemental concentration of geophagy clay and evaluates its adsorptive capacity in removing Cd^2+^ and Pb^2+^ in water. Fifteen clay samples were randomly collected from three layers in the space of one meter apart from Amawom clay deposit in Ikwuano local government, Southeast Nigeria. Elemental analysis was carried out using the inductively coupled plasma mass spectrophotometer (ICP-MS), and chemical characterization was performed with Fourier transform infrared spectroscopy (FT-IR) and X-ray diffractometer (XRD). The adsorptive capacity of Cd^2+^ and Pb^2+^ on the clay samples was evaluated using standard solutions of the metal ions. The result of the elemental analysis in mg/kg (Pb ≤ 12.4, Zn ≤ 2.75, Co ≤ 1.50, Ni ≤ 1.47, Mn ≤ 15.0, Cd = 0.01, Ca ≤ 300, Al ≤ 3466, Na ≤ 13.3, and Hg = 0.02; *P* ≤ 40.0) revealed that the concentrations of most of the studied metals in the three layers are statistically similar and fall below the permissible recommended safety levels. The presence of functional groups (hydroxyl, amine, and carboxylic/ester) and minerals (kaolinite, goethite, and quartz) provided evidence of the good adsorptive properties of the clay samples. The adsorption of Cd^2+^ and Pb^2+^ unto the clay samples increased with increase in pH, concentration, time, and temperature, and the equilibrium data for the adsorption fitted well into Langmuir isotherm. The study, therefore, concluded that geophagy clay possesses the capacity to adsorb Cd^2+^ and Pb^2+^ for water treatment.

## 1. Introduction

The act of eating earthly material such as clay, i.e., geophagy, especially among pregnant women and children in achieving and maintaining human health, have been a common practise in many countries in the world [1–4]. Geophagy clay has versatile use because of its low cost, abundance, high sorption potential for ion exchange, environment-friendly nature, and a wide pH range [5–7]. It has been used topically in mud spas (pelotherapy) to adsorb toxins from skin, remove oils, secretions, and contaminants and provide heat to stimulate circulation for rheumatism treatment [8]. Geophagy clay has been reported to have the ability to absorb dietary and bacterial toxins associated with gastrointestinal disturbance [5, 6], free radicals, and pesticides from the gastrointestinal tract. Generally, clay as a very good adsorbent has been widely used for the removal of contaminants ranging from metals to priority pollutants from contaminated drinking water and its sources [7, 9].

Clay properties including high adsorption and absorption capacities, cation exchange capacity, and extremely fine particle size, e.g., smectites (expandable clay minerals) and kaolin group minerals and structural/surface (resistance to wear and resistance to chemical attack) properties. These properties permit their use in removing oils, secretions, toxins, and contaminants from materials. The study of the mineralogical composition of geophagy clay has predominantly indicated the presence of kaolinite with minor palygorskite, nontronite, illite, K-feldspar, halloysite, and calcite [3, 10, 11].

Despite the knowledge of the adsorptive properties of geophagy clay with its mineralogical content, its use in adsorption of heavy metals in environmental samples has not been well documented [3, 4, 10]. This study, therefore, evaluates the contaminant level and adsorptive capacity of geophagy clay for the removal of heavy metals in water. Pb^2+^ and Cd^2+^ were selected for the adsorption study because of their hazardous and toxic nature; they commonly occur at high concentrations in contaminated waters. They are carcinogen and can cause kidney damage and renal disorder [7, 12]. The adsorption capacity of the clay sample was evaluated by studying the experimental parameters, which affect the removal process such as initial metal ion concentration, pH, sorbent dosage, and contact time. The experimental data were fitted using Langmuir and Freundlich isotherm models to determine the adsorption isotherm. Also, the mechanism of the adsorption was studied by fitting the experimental kinetic data using pseudo-first order and pseudo-second order kinetic models.

## 2. Materials and Methods

### 2.1. Sample Collection

The study site was a clay deposit located at Amawom in Ikwuano local government, Southeast Nigeria. Ikwuano is located between latitudes 5°24′–5°29^1^ N and longitudes 7°32′–7°37′ E. It falls in typical rainforest vegetation in the southeast agroecological zone of Nigeria with typology of the degraded humid forest ecology in the sub-Saharan Africa [13]. The soils are coarse-textured, deep, and well-drained. They have weak, coarse, and fine crumbs at the epipedon and underlain by moderate subangular blocky structures in the endopedon. The soils are characterized by toposequence and lithosequence and are classified as Ultisols because the base saturation is <25.0% below 120 metre depth [14]. The sampling site was stratified into three layers of approximately one metre apart. Five samples were randomly collected from each layer to make a total of fifteen samples. The collected clay samples were transported to the laboratory where they were air-dried for fourteen days at ambient temperature and pulverized prior to analysis.

### 2.2. Elemental Analysis

Preliminary elemental study of the collected clay samples was carried out in duplicate. Total acid digestion with aqua regia (2 ml HNO_3_ + 6 ml HCl) was carried out, and the concentrations of elements in the digested solutions were measured using an inductively coupled plasma mass spectrophotometer (ICP-MS) (Perkin Elmer Nexion 300 Q ICP-MS).

### 2.3. Characterization

#### 2.3.1. Characterization of the Functional Group

Fourier transform infrared spectrophotometer (FT-IR) was used to determine the functional group on the clay samples, which serves as binding sites for adsorption. 2 mg of the samples were grounded and mixed uniformly with 200 mg pure KBr powder. The IR spectra of clay samples were run as KBR pellets on the FT-IR system (Spectrum BX PerkinElmer, England) in the frequency range 350–4000 cm^−1^.

#### 2.3.2. Mineralogical Characterization

X-ray diffractometer (XRD) was used to determine the minerals present in the clay samples. The clay sample was milled using a McCrone grinding mill with agate grinding elements in a jar. The device is equipped with a Ni filter and generates monochromated Cu-K*α* radiation (*λ* = 0.154 nm) operated at 40 kV accelerating voltage and 30 mA current. The samples were scanned in step mode with a 2°min^−1^ scan rate in 2*θ* range of 5–65°.

#### 2.3.3. Adsorption Studies of the Clay Samples

Clay samples from the topmost (clay-1) and bottom layers (clay-2) were selected for the adsorption study. These two layers were selected because most of the properties of the middle clay samples were similar to either the topmost or bottom layer. Their mineralogy indicated some minerals which were not found in the middle layer. Adsorption study of Pb^2+^ and Cd^2+^ on these clay samples was carried out in duplicate using the batch technique at room temperature. The batch mode was selected because of its simplicity and reliability. In this study, the effect of pH from 3 to 9 (the pH of the solution was adjusted with dilute HCl or NaOH solution), concentration range from 10–60 mg/L, and contact time in the range of 30–120 min were evaluated. The percentage adsorption was then calculated by subtracting final concentration from initial concentration.

#### 2.3.4. Adsorption Isotherms

The adsorption isotherms for Pb (II) and Cd (II) removal were evaluated with Langmuir and Freundlich isotherm models at pH of 5, 0.1 g of clay samples, and concentrations of metal ions (10, 30, 40, and 60 mg/l). Langmuir and Freundlich isotherm models were fitted to the adsorption data, and their constants were evaluated. The Langmuir model is represented by equation (1), where Ce and qe are the initial concentration of the adsorbate and the amount absorbed at equilibrium, respectively, and *b* and qm are Langmuir coefficients representing the equilibrium constant for the adsorbate–adsorbent equilibrium and the monolayer capacity. Equation (2) represents the Freundlich model where KF (mg g^−1^) and *n* are Freundlich constants incorporating all factors affecting the adsorption process such as adsorption capacity and intensity of adsorption. These constants are determined from the intercept and slope of linear plot of logqe versus logCe, respectively [15]:

(1) $$\begin{array}{c} \frac{\mathrm{Ce}}{\mathrm{qe}}=\frac{1}{b\mathrm{qm}}+\frac{1}{\mathrm{qm}}\mathrm{Ce}, \end{array}$$

(2) $$\begin{array}{c} \log\;\mathrm{qe}=\mathrm{logKF}+\left(\ln\right)\log\mathrm{Ce}. \end{array}$$

### 2.4. Quality Control

Analytical grade metal salts Pb (NO_3_)_2_ and Cd (NO_3_)_2_ 4H_2_O were used without further purification. Stock solutions (1000 mg/l) of Pb (NO_3_)_2_ and Cd (NO_3_)_2_ 4H_2_O were prepared in distilled water. The stock solutions were diluted as required to obtain standard solutions containing 10, 30, 40, and 60 mg/l of Pb^2+^ and Cd^2+^. All the instruments were calibrated before use to ensure accurate results.

## 3. Results and Discussion

### 3.1. Result of Elemental Analysis

The result of the elemental analysis of the clay samples is given in Table 1. Elemental analysis was performed to evaluate the concentrations of essential and nonessential elements present in the sample. The result shows the presence of essential (Ca, Na, and P) and nonessential elements ( Pb, Cd, Al, and Hg), which vary at different layers. The highest mean concentration of Pb, Ni, Co, Fe, P, Al, and Hg was detected at the bottom layer of the clay sample while the minimum values were recorded at the top layer except for mercury. Zn and Na have their highest mean concentration at the middle layer while the mean concentrations at the top and bottom layer were statistically similar (Table 1). Ca has the highest value at the top layer while the minimum values are at the middle and bottom layer.

The levels of Pb, Ni, Co, Fe, P, and Al in the clay samples collected from the three depths follows this trend: top < middle < bottom. The statistical difference in the levels of Cd, P, Na, and Hg found in the three layers were not significant at *P* < 0.05. The levels of Cd, Pb, Zn, Fe, and Mn in all clay samples were compared with the permissible level (WHO) of these metals in food and soil, and the concentrations of all the metals were found to be significantly lower than the set standards at (*P* < 0.05) (Table 1).This result is not in agreement with the results of Lar et al. [16], Bonglaisin et al. [17], and Bonglaisin et al. [11] who recorded higher levels of Pb, Zn, Hg, and As in geophagy clay samples. Manganese (Mn) and iron (Fe) are essential constituents of human diet at low concentrations, and it is required for normal metabolism of amino acids, lipids, proteins, and carbohydrates. Fe is considered as an essential mineral because it is required for the synthesis of the oxygen-carrying proteins (haemoglobin) [18].

### 3.2. Result of Functional Groups Characterization Using FT-IR

The FT-IR analysis of the clay samples was carried out to determine the functional groups on the surface of the clay; functional groups act as binding sites for metal adsorption. Peaks with similar functional groups were observed in the three layers. The strong sharp bands observed in the three layers between 3600 and 3700 cm^−1^ are due to stretching vibration of surface–OH groups and adsorbed water molecules with bending vibration mode around 1637 cm^−1^ [7, 19]. NH (1° and 2° amines) at 748–792 cm^−1^, strong CO (acid/ester/ether) between 1005 and 1116 cm^−1^, CN (aliphatic amines) between 1024 and 1032 cm^−1^, and CBr (alkyl halide) between 518 and 690 cm^−1^. This result is similar to the findings of Njoya et al. [20], Dawodu and Akpomie [21], and Burham and Sayed [7]. A typical peak observed at 1109 cm^−1^ indicates the presence of calcite as carbonate mineral [22]

Also, the OHs of hydroxyl basal surfaces of kaolin interact with positively charged metal cations via H-bonding during adsorption of metals [19]. The heteroatoms (OH (alcohol and acid), CO (acid/ester/ether), CN, and NH) on the functional groups of the clay with lone pair of electrons serve as binding sites for the removal of both inorganic (heavy metals) and organic contaminants (dye and paints) in water.

### 3.3. Result of Mineralogical Composition

**Figure 1:**
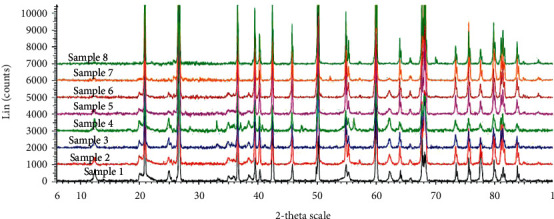
XRD spectra of top (samples 1, 4, and 7), middle (samples 2, 5, and 8) and bottom (samples 3 and 6) clay samples.

The mineralogical phase in the spectrum of all the analysed clay samples were similar and were dominated by the clay mineral (kaolinite (Al_2_Si_2_O_5_ (OH)_4_)) and quartz (SiO_2_). This is similar to the report of Okunlola and Owoyemi [3] for geophagy clay samples from southern part of Nigeria. Sample 4 from top layer also indicated the presence of pyrite (FeS_2_), and sample 3 from bottom layer indicated the presence of goethite (FeO (OH) (Figure 1). Kaolinite is the dominant clay mineral that was found in the three layers with X-ray diffraction pattern at 2*θ* = 36° and a d-spacing of 1.31 nm (Figure 1). Quartz is a dominant mineral from the bulk clay sample with an X-ray diffraction pattern at 2*θ* = 20°–70° and d-spacing 2.36 nm. The mineralogical phase also suggests goethite and pyrite with a X-ray diffraction pattern at 2*θ* = 25° and 21.2° with d-spacing values of 4.26 and 4.98 indicated in bottom and top layer clay samples. The presence kaolinite, goethite, and pyrite in the clay sample may enhance its adsorptive capacity for both organic and inorganic contaminants in the environment [7, 23]. Isomorphous substitution of Si^4+^ by Al^3+^ in surface tetrahedral sheets on the clay gives small negative charge of siloxane faces responsible for adsorption/exchange of cations such as metallic ions.

Clay mineral (kaolin) has a negative surface charge and high surface area, which is an advantage for the adsorption of heavy metals from the solution [24, 25]. The mineralogy of the studied geophagy clay is similar to the findings of Ekosse et al. [26].

### 3.4. Evaluation of the Adsorptive Capacity of the Clay Material

**Figure 2:**
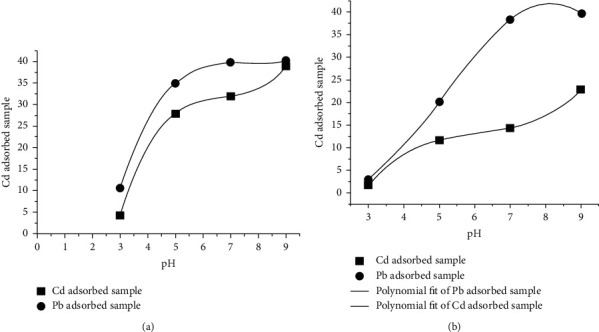
Graph of concentration of Cd (II) and Pb (II) adsorbed by clay-1 (a) and clay-2 (b) on *y*-axis against pH on *x*-axis.

**Figure 3:**
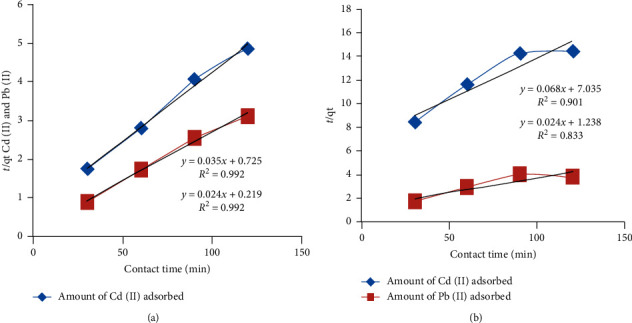
Graph of time dependent of Cd^2+^ (blue) and Pb^2+^ (red) on clay-1 (a) and clay-2 (b) (2^nd^ order).

**Figure 4:**
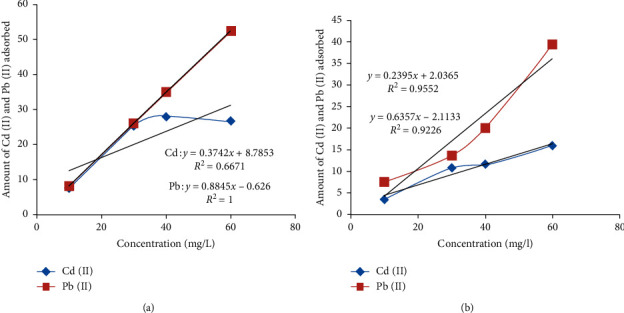
Graph of Cd (II) and Pb (II) adsorption on clay-1 (a) and clay-2 (b) at varying concentrations.

**Figure 5:**
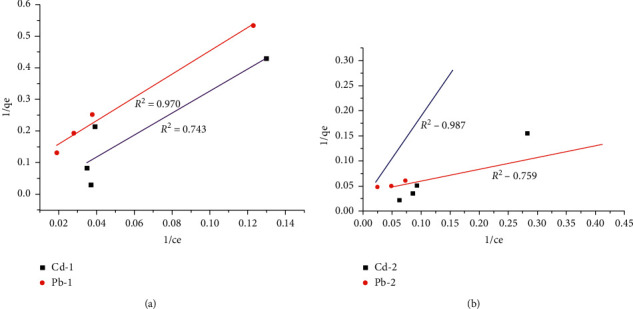
Langmuir isotherm for Cd (II) and Pb (II) adsorption on clay-1 (a) and clay-2 (b).

**Figure 6:**
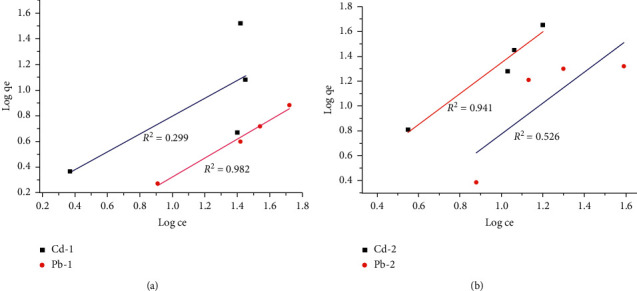
Freundlich isotherm for Cd (II) and Pb (II) adsorption on clay-1 (a) and clay-2 (b).

**Figure 7:**
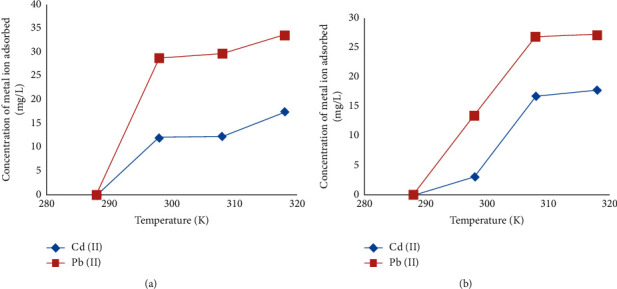
Effect of solution temperature on the amount of metal ion adsorbed for clay-1 (a) and clay-2 (b).

The adsorptive capacity of the two clay samples was evaluated using standard solution of Pb^2+^ and Cd^2+^. The effects of contact time, concentration of adsorptive solutions, temperature, and pH of the solutions were studied. The results are illustrated graphically in Figures 2–7.

#### 3.4.1. Effect of Change in pH on the Adsorption Capacity of the Clay Samples

The pH of aqueous solution is an important variable, which controls the adsorption of metal at the clay–water interface level [27]. As the acidity of the medium decreases from 3 to 5, the extent of adsorption (%) as well as the amount adsorbed (qe) increases. The adsorption efficiency of Pb ^2+^ increased significantly as pH increases (Figure 2), similar to the report of Yin et al. [25].

The rate at which Pb^2+^ is adsorbed on the two clay samples is greater than that of Cd^2+^, and clay-1 adsorb the metal more than clay-2 (Figure 2). The negative surface charge of clay in solution affects the pH and sorption of charged species on the clay surface [28, 29]. According to El-Maghrabi and Sara [27], at low pH, competition exists between the positively charged hydrogen ions and metal ions for the available adsorption sites on the negatively charged clay surface. As the pH increases, more of the positively charged metals ions in solution are adsorbed to an extent on the negative clay surface, and thus the percentage removal of the metal ions increases as observed in this study [29].

#### 3.4.2. Adsorption Kinetics: Pseudo-Second Order Model

The pseudo-second order model was used to evaluate the effect of contact time on the adsorption of Cd and Pb ions on the studied clay samples. This was obtained from the evaluation of kinetic data using the following equation:t/qt = 1/k_2_q^2^e +(1/qe)t

(3) $$\begin{array}{c} \frac{t}{\mathrm{qt}}=\frac{1}{k_{2}q^{2}e}+\frac{1}{\mathrm{qe}}t, \end{array}$$

where *k*_2_ (g·mg^−1^·min^−1^) is the second order rate constant, *k*_2_*q*^2^*e* is known as the initial sorption rate, and qe and qt (mg/g) represent the amount of Cd (II) or Pb (II) adsorbed (mg/g) at equilibrium and at time *t* (min.), respectively. (*t*/qt) was plotted against contact time, and values of the second order rate constant (*k*_2_) and the equilibrium adsorption capacity (qe) were calculated from the intercept and slope of the plot.

The experimental data fit very well to this model with very high correlation coefficients, *R*^2^ > 0.901 for cadmium and *R*^2^ > 0.833 for lead (Figure 3). This was found to be more appropriate for the description of the pseudo-second order model for adsorption, and the finding is in agreement with the results of Ulmanu et al. [30], who investigated heavy metals removal from aqueous solution using peat, El-Maghrabi and Sara [27], and Burham and Sayed [7].

#### 3.4.3. Effects of Initial Metal Concentration

The plot of the amount of metal adsorbed against the initial metal ion concentration shows that the amount of metal adsorbed by the clay increases with increasing metal concentration (Figure 4). The result of this finding is similar to that of Mohammed et al. [31] where phosphate modified with kaolinite clay was use to adsorb Pb (II), Zn (II), and Cd (II).

Meanwhile, at low initial concentration, the rate of adsorption was low, but as the concentration increased, adsorption of the metal by the clay also increased. It is obvious that, for higher initial concentration, more efficient utilization of sorption sites is expected due to a greater driving force by a higher concentration gradient. The result shows that Pb^2+^ is well adsorbed than Cd^2+^ in the two clay samples, which is similar to the findings of Mohammad et al. [31].

#### 3.4.4. Adsorption Isotherms

The adsorption isotherms for Pb^2+^ and Cd^2+^ removal were evaluated, and the data were fitted to Langmuir and Freundlich isotherm. The data obtained for clay-1and clay-2 fitted well to Langmuir isotherm compared to Freundlich isotherm (Figures 5 and 6). Satisfactory conformity between experimental data and the model-predicted values was expressed by the correlation coefficient (*R*^2^).

The Langmuir model assumes homogenous adsorbent surface and monolayer adsorption, and the adsorption energy is uniform in all sites while the Freundlich model assumes heterogeneous adsorbent surface, and that strong binding sites are occupied first, until adsorption energy is exponentially decreased upon the completion of adsorption process [15]. The Freundlich model has been found to be the most appropriate to describe the adsorption of different adsorbates from aqueous solutions for two parameter monolayer adsorption isotherm models. Pb (II) and Cd (II) were adsorbed well on clay-2 than on clay-1. Pb adsorption is well fitted to Langmuir and Freundlich isotherm than Cd adsorption. Adsorption of the two metals fitted well to the Langmuir model better than Freundlich isotherm on either clay-1 or clay-2 (Figures 5 and 6). This suggests that the adsorption of the two metals on the clay samples is a homogenous and monolayer adsorption. This is in agreement with the results reported by Elkosse et al. [26], Matlok et al. [19], and Djebbar [32].

#### 3.4.5. Thermodynamics Studies

The adsorption of Pb^2+^ and Cd^2+^ unto clay at different temperatures showed an increase in the adsorption capacity with increase in temperature (Figure 7) indicating that the adsorption process is a chemical adsorption rather than a physical one [33], and this suggests that the adsorption is an endothermic one, which corroborates with the findings of Akpomie et al. [34] and Al-Essa and Khalili [29].This may be due to the fact that Pb^2+^ and Cd^2+^ ions gained more kinetic energy to diffuse from the bulk phase to the solid phase with an increase in solution temperature [34].

The feasibility of the adsorption process was evaluated, and thermodynamic parameters such as the standard free energy (Δ*G*^0^), enthalpy change (Δ*H*^0^), and entropy change (Δ*S*^0^) were calculated from the slope and intercept of the plot lnKc versus (1/*T*) (temperature K^−1^) and Δ*G*^0^=RT  ln  *K*_*d*_(*K*_*d*_ − equilibrium distribution constant). The result of the enthalpy (Δ*H*^0^) and entropy change (Δ*S*^0^) is presented in Table 2. The negative values of Δ*G*^0^ obtained at all temperatures for both metals ion are an indication that the adsorption is spontaneous in nature (Table 2). It was also observed that the change in free energy increases with increase in temperature.

## 4. Conclusions

5.0 assessment of the elemental concentration and chemical characterization of geophagy clay and its adsorptive capacity in removing Cd^2+^ and Pb^2+^ in water was evaluated. The concentrations of most of the studied metals in the three layers are statistically similar and fall below the permissible safety levels. The study geophagy clay was found to possess unique surface functional groups and minerals, which made it a good adsorbent for Cd^2+^ and Pb^2+^, and this can be adopted in water treatment.

## Figures and Tables

**Table 1:** Results of metal analysis of the studied clay (mg/kg).

	Top layer	Middle	Bottom	Food (WHO)	Soil (WHO)
Pb	6.33 ± 0.54^ab^	7.70 ± 0.47^b^	12.4 ± 1.45^c^	0.3	25
Zn	2.00 ± 0.20^a^	2.75 ± 0.46^b^	1.90 ± 0.20^a^	300	100
Ni	0.86 ± 0.06^ab^	0.93 ± 0.21^ab^	1.47 ± 0.51^b^		
Co	0.23 ± 0.06^a^	0.43 ± 0.12^a^	1.50 ± 0.89^b^		
Mn	15.0 ± 0.00^b^	13.3 ± 2.04^ab^	11.0 ± 2.00^a^	500	2000
Fe	4366 ± 245^ab^	4400 ± 140^ab^	15233 ± 238^b^	425	50000
Cd	0.01 ± 0.00	0.01 ± 0.00	0.01 ± 0.00	0.1	3
Ca	300 ± 100^b^	100 ± 0.00^a^	100 ± 0.00^a^		
P	26.7 ± 11.5^a^	30.0 ± 10^a^	40.0 ± 0.00^a^		
Al	3466 ± 611^ab^	4166 ± 416^b^	5333 ± 513^c^		
Na	10.0 ± 0.00^a^	13.3 ± 5.77^a^	10.0 ± 0.00^a^		
Hg	0.02 ± 0.01^ab^	0.02 ± 0.00^b^	0.02 ± 0.002^b^		

Values carrying different superscripts along the same row differ significantly (*P* < 0.05).

**Table 2:** Adsorption thermodynamics parameters for the adsorption of Cd^2+^ and Pb^2+^ by clay-1 and clay-2.

Clay	Metal ion	Temp (K)	Gibb's free energy (J/mol)	Δ*H*° (KJ mol^−1^)	Δ*S*°(JK^−1 ^mol^−1^)
1	Cd^**2+**^	298	−2123.193	341	−99.1
		308	−2111.598		
		318	−656.789		
	Pb^**2+**^	298	2339.263	4196	−73.3
		308	2725.137		
		318	4322.052		
2	Cd^**2+**^	298	−6217.108	1112	−0.213
		308	−848.772		
		318	−585.724		
	Pb^**2+**^	298	−1662.206	1059	−0.249
		308	1826.683		
		318	1984.941		

## Data Availability

The data used to support this study are included within this article.
